# Standard: Human intestinal cancer organoids

**DOI:** 10.1186/s13619-023-00167-6

**Published:** 2023-06-28

**Authors:** Hanqing Lin, Yalong Wang, Chunyan Cheng, Yuxin Qian, Jie Hao, Zhen Zhang, Weiqi Sheng, Linhong Song, Chu-Xia Deng, Bing Zhao, Jiani Cao, Lei Wang, Liu Wang, Lingmin Liang, Wenli Kelly Chen, Chunping Yu, Zhijian Sun, Yingying Yang, Changlin Wang, Yong Zhang, Qiyuan Li, Ka Li, Aijin Ma, Tongbiao Zhao, Ye-Guang Chen, Guoqiang Hua

**Affiliations:** 1 D1Med Technology (Shanghai) Inc, Shanghai, 201802 China; 2 The State Key Laboratory of Membrane Biology, Tsinghua-Peking Center for Life Sciences, School of Life Sciences, Tsinghua University, Beijing, 100084 China; 3 Guangzhou Laboratory, Guangzhou, 510005 China; 4 Guangzhou Institutes of Biomedicine and Health, Chinese Academy of Sciences, Guangzhou, 510530 China; 5 Guangzhou Hua Yi Regeneration Technology Co., Ltd, Huangpu District, Guangzhou, 510700 China; 6 National Stem Cell Resource Center, State Key Laboratory of Stem Cell and Reproductive Biology, Institute for Stem Cell and Regeneration, Institute of Zoology, Chinese Academy of Sciences, Beijing, 100101 China; 7 Beijing Institute for Stem Cell and Regenerative Medicine, Beijing, 100101 China; 8 University of Chinese Academy of Sciences, Beijing, 100049 China; 9 Institute of Clinical Science, Zhongshan Hospital, Fudan University, Shanghai, 200433 China; 10 Department of Radiation Oncology and Cancer Institute, Fudan University Shanghai Cancer Center Fudan University, Shanghai, 200032 China; 11 Department of Oncology, Shanghai Medical College, Fudan University, Shanghai, 200032 China; 12 Shanghai Key Laboratory of Radiation Oncology, Shanghai, 200032 China; 13 Department of Pathology, Fudan University Shanghai Cancer Center, Shanghai, 200032 China; 14 Sichuan Academy of Medical Sciences & Sichuan Provincial People’s Hospital, Chengdu, 610072 China; 15 Cancer Centre, Faculty of Health Sciences, University of Macau, Macau, 999078 SAR China; 16 State Key Laboratory of Genetic Engineering, School of Life Sciences, Fudan University, Shanghai, 200438 China; 17 China Innovation Center of Roche, Li Shi Zhen Road, Pudong, Shanghai, 201203 China; 18 Eli Lilly and Company, Pudong, Shanghai, 201203 China; 19 K2 Oncology Co., Ltd, KeChuang Street, Beijing, 100176 China; 20 Qilu Pharmaceutical Co., Ltd, Jinan, 250104 China; 21 China National Institute of Standardization, Beijing, 100191 China; 22 Chinese Society for Stem Cell Research, Shanghai, 200032 China; 23 HHLIFE Co., Inc, Shenzhen, 518040 China; 24 China National GeneBank, Shenzhen, 518000 China; 25 Beijing Technology and Business University, Beijing, 100048 China; 26 School of Basic Medicine, Jiangxi Medical College, Nanchang University, Nanchang, 330031 China

## Abstract

Intestinal cancer is one of the most frequent and lethal types of cancer. Modeling intestinal cancer using organoids has emerged in the last decade. Human intestinal cancer organoids are physiologically relevant in vitro models, which provides an unprecedented opportunity for fundamental and applied research in colorectal cancer. “Human intestinal cancer organoids” is the first set of guidelines on human intestinal organoids in China, jointly drafted and agreed by the experts from the Chinese Society for Cell Biology and its branch society: the Chinese Society for Stem Cell Research. This standard specifies terms and definitions, technical requirements, test methods for human intestinal cancer organoids, which apply to the production and quality control during the process of manufacturing and testing of human intestinal cancer organoids. It was released by the Chinese Society for Cell Biology on 24 September 2022. We hope that the publication of this standard will guide institutional establishment, acceptance and execution of proper practocal protocols, and accelerate the international standardization of human intestinal cancer organoids for clinical development and therapeutic applications.

## Scope

This document specifies the ethical requirements, technical requirements, and testing methods for human intestinal cancer organoids.

This standard applies to the production and testing of human intestinal cancer organoids.

## Normative references

The following referenced documents are indispensable for the application of these documents. For dated references, only the edition cited applies. For undated references, the latest edition of the referenced document (including all amendments) applies.

- WS 213 Diagnosis for Hepatitis C
- WS 293 Diagnostic Criteria for HIV/AIDS
- WS 299 Diagnostic Criteria for Viral Hepatitis B
- Pharmacopoeia of the People’s Republic of China (2020 Edition)
- National Guide to Clinical Laboratory Procedures

## Terms and definitions

The following terms and definitions apply to this document.

### Organoids

Three-dimensional (3D) structures that grow from stem cells or progenitor cells in vitro*,* consist of organ-specific cell types, and can mimic the in vivo architecture and specific function of original tissue (Clevers 2016; Fujii and Sato 2021; Kim et al. 2020; Sato et al. 2009).

### Tumor organoids

Organoids that derived from tumor cells from tumor tissues or other specimens containing tumor cells from patients and cultured in vitro, and can expand and recapitulate tumor pathologic features, genetic characteristics, and treatment response (Fujii et al. 2016; Kolahi et al. 2020; Tuveson and Clevers 2019).

### Human intestinal cancer organoids

Organoids that develop from intestinal tumor cells of patients with a pathological diagnosis of intestinal cancer, and can simulate the characteristics of intestinal cancer (Mo et al. 2022; Sato et al. 2011; van de Wetering et al. 2015).

### Passage

Process of dissociating existing organoids into smaller fragments, or single cell via physical, chemical, or biological methods, and keeping them growing in vitro under the same culture conditions (Ganesh et al. 2019).

### Cryopreservation

Freezing process by which organoids are maintained at low temperature in an inactive state for maintaining cellular composition, gene expression, and functional properties.

### Thawing

Process of bringing frozen organoids from an inactive to an actively growing state.

## Ethics requirements

A legal and valid informed consent shall be signed by the donor who provides the tissue to develop the organoid. The consent form includes, but not limited to, potential research and therapeutic applications under the appropriate conditions, potential commercial applications of research results, and other issues applicable.

The production and research project of human intestinal cancer organoids shall be approved by the ethics review committee.

The personal information of donors shall be protected.

## Technical requirements

### Morphology

Human intestinal cancer organoids shall have a clear edge and cytoplasm when observed under the optical microscope. They shall be formed by cell clusters, exhibiting compact, loose, cystic, or mixed morphology (Betge et al. 2022; van de Wetering et al. 2015; Wang et al. 2022; Yao et al. 2020).

### Culture and growth

The first generation of human intestinal cancer organoids developed from tissue or cells from patients, shall be kept alive in vitro for at least two months and shall be passaged for at least three generations (Lv et al. 2023; Sato et al. 2011).

Post-passage organoids shall be reconstructed in vitro into new analogous organoids, and their morphology and characteristics shall be consistent with those of the pre-passage organoids (Sato et al. 2011).

### Viability

The organoid viability shall be ≥ 50% after thawing, and these living organoids shall be subcultured in vitro (Lv et al. 2023).

### Microorganisms

Organoids shall be negative for fungi, bacteria, HBV, HCV, HIV, and exogenous viral factors.

### Identity

The identity of organoids shall match that of the donor tissue by STR analysis (Lee et al. 2015).

### Pathological features

Pathological features of organoids shall be recognized by qualified pathologists. These features shall be consistent with the typical features of tumor cells based on H&E staining images, such as hyperchromasia, abnormal mitosis, imbalanced nucleocytoplasmic ratio, etc. (De Angelis et al. 2022).

The immunohistochemical marker proteins CDX2 and CK20 of differentiated intestinal adenocarcinoma organoids shall be positive and the distribution of expression shall be non-polar and disordered (De Angelis et al. 2022; Ganesh et al. 2019).

### Genetic characteristics

Genetic variant testing shall be performed on the organoids, and the test results shall be consistent with the results of the original tumor tissue. The tested genes shall include, but not limited to, *KRAS, NRAS, HRAS, BRAF, APC, TP53*, *SMAD4*, etc (Fujii et al. 2016; Ganesh et al. 2019; van de Wetering et al. 2015; Wanigasooriya et al. 2022; Zhao et al. 2022).

## Test methods

### Morphology

Observe organoid morphology by the inverted phase contrast microscope.

### Quantity

Count the organoid number, defined by a pre-defined diameter threshold, from the images taken by an inverted phase contrast microscope that is attached with a scale bar.

### Viability

Organoid viability testing shall be performed on the primary organoids and passaged organoids, and the method in Appendix A shall be followed.

### Microorganisms

#### Bacteria and fungi

The “1101 Sterility Inspection Method” in *Pharmacopoeia of the People's Republic of China* (2020 edition) shall be followed.

#### Mycoplasma

The “3301 Mycoplasma Inspection Method” in *Pharmacopoeia of the People's Republic of China* (2020 edition) shall be followed.

#### HIV

The method in WS 293 shall be followed.

#### HBV

The method in WS 299 shall be followed.

#### HCV

The method in WS 213 shall be followed.

#### Exogenous viral factors

The “3302 Exogenous Viral Factors Inspection Method” in *Pharmacopoeia of the People's Republic of China* (2020 edition) shall be followed.

### STR

The method in Appendix B shall be followed.

### Pathological features

The method in Appendix C shall be followed.

### Genetic characteristics

The method in Appendix D shall be followed.

## Data Availability

Not applicable.

## Appendix A

Normative Appendix: Organoid Viability Test (Calcein-AM Staining Method)

**Instruments**

Inverted microscope

Fluorescence microscope

**Reagents**

Unless otherwise specified, the reagents used shall be analytically pure, and the water used for testing shall be deionized water.

Dimethyl sulfoxide (DMSO) for cell culture

Phosphate-buffered saline (PBS): pH 7.4

Storage of Calcein-AM solution: 2 mmol/L in DMSO

**Testing protocol**

Organoid counting

Place the organoids under the microscope to observe their morphology and status. Determine whether the organoid morphology meets the requirements of 6.1 by visual observation, and count the organoids with a diameter ≥ 20 μm.

Living organoid counting

Add the Calcein-AM storage solution to the medium to a final concentration of 0.2 μmol/L, and incubate the mixture at 37℃ for 60 min. Then remove the medium with Calcein-AM gently with PBS and add fresh medium. The organoids are observed and photographed by fluorescence microscope at 490 nm excitation wavelength and 515 nm emission wavelength. Living organoids are in green with clear edges. Then count the living organoids ≥ 20 μm in diameter.

**Organoid counting**

Tomographically scan the organoids using a microscope and image acquisition software, with the interlayer height set to the range of 10 μm to 200 μm. Superimpose the scanned images to be a single planar map, and then count the organoids in the final map.

**Organoid viability**

Organoid viability is calculated according to Eq. (A.1):

A.1 $$X={N}_{\text{alive}}/{N}_{\text{total}}\times 100\%$$

In this equation:

*X* —Organoid viability,

*N*_alive_—Number of living organoids,

*N*_total_—Total number of organoids.

**Calculation and analysis**

Repeat the procedure twice more according to A.3, calculate the average of the three living organoid ratio results, and record it as the organoid viability.

**Accuracy**

The absolute difference between the results of three independent determinations obtained under reproducible conditions shall not exceed 10% of the arithmetic mean.

## Appendix B

Normative Appendix: Organoid Authentication by STR Profiling

Instruments

Centrifuge

PCR-Cycler

Electrophoresis apparatus

**Reagents**

Cell DNA extraction kit

STR DNA profiling kit

**Sample storage**

The samples are prepared and stored below -80℃.

**Testing protocol**

Sample preparation

The organoids are cultured in the Matrigel to a stable growth state, and then they are mechanically pipetted out of the Matrigel. The mixture is collected in a centrifugal tube, the organoids are collected by centrifugation, and the supernatant is discarded.

Extraction of DNA

A) Perform genomic DNA extraction from organoids and primary tumor tissues according to the instructions of the cellular DNA extraction kit.
B) Measure the absorbance of extracted DNA by UV spectrophotometer to ensure that the ratio of A260/A280 is between 1.8 and 2.0.
C) DNA volume ≥ 20 μL, DNA concentration ≥ 50 ng/μL.

PCR amplification

A) Perform STR DNA amplification according to standard PCR amplification methods or the commercially approved kit instructions.
B) Set up a negative control group, a sample detection group, and a positive control group. Use sterile water as the template for PCR amplification in the negative control group; use the DNA extracted from organoid and primary tumor tissue samples as a template for PCR amplification in the sample detection group; use the DNA template for amplification in the positive control group.
C) Detect the PCR products of three groups by agarose gel electrophoresis. Clear target band shall be observed in the positive control but not in the negative control.

STR genotyping

Detect PCR products by capillary electrophoresis gene analyzer and STR genetic map data are obtained. The PCR banding pattern of organoids and primary tumor tissue shall be consistent.

**Results analysis**

When STR alleles contain the same number of repeats, only one allele peak shall appear in the profile, when they contain different numbers of repeats, two allele peaks appear in the profile. The test is considered valid when no allele peaks appeared in the negative control group and the positive control group is consistent with its standard genotyping data.

If more than two allelic peaks are present at the STR locus of the tested sample, the sample shall be determined to be cross-contaminated after repeated experiments to exclude interfering factors such as mutations in the primer binding region, provided that the test is valid.

## Appendix C

Normative Appendix: Organoid histopathology testing (Paraffin Embedding Method)

**Instruments**

Paraffin-embedding machine

Paraffin-slicer

**Reagents**

Unless otherwise specified, the reagents used shall be analytically pure, and the water used for testing shall be deionized water.

Paraffin section preparation reagents: prepare reagents required for paraffin embedding according to the corresponding requirements, including fixing solution, dehydration solution, paraffin, dewaxing solution, rehydration solution, ethanol, xylene, and neutral resin.

H&E staining reagent: hematoxylin, eosin.

Immunohistochemical staining reagents: prepare antigen repair solution, primary antibody, secondary antibody, closure solution, PBS, DBA according to the corresponding requirements.

**Testing protocol**

Sample preparation and fixation

The organoids are mechanically pipetted out of Matrigel gently and transferred to a 15 mL centrifuge tube. Collect the organoids by centrifugation and discard the supernatant. The organoids are fixed with 4% paraformaldehyde for 15–30 min.

Paraffin section preparation of organoids

The organoid samples are fixed, dehydrated, hyalinized, immersed in paraffin, and embedded with a paraffin-embedding machine according to the method of paraffin section. Cut into standard thickness with paraffin-slicer.

H&E staining

Paraffin sections of organoids are dewaxed, rehydrated, stained with hematoxylin and eosin, then dehydrated with ethanol, hyalinized by xylene, and sealed by neutral resin.

Immunohistochemistry staining

Paraffin sections of organoids are dewaxed, rehydrated, antigen repaired and sealed with blocking solution, sections are incubated with primary antibody and then cleaned with PBS, sections are followed by incubated with second antibody and cleaned with PBS. Perform the color reaction with DAB and add water to stop the color reaction. The sections are lining dyed by hematoxylin, hyalinized with hydrochloric acid and flushing. Then dehydrated with ethanol, hyalinized by xylene, and sealed by neutral resin.

**Results analysis**

The test results obtained are analyzed and judged by personnel qualified in the pathological diagnosis, and these results shall be consistent with the results of the original tumor tissue.

## Appendix D

Normative Appendix: Organoid Gene Mutation Testing

**Instruments**

Centrifuge

**Reagents**

Prepare the Cell DNA extraction kit according to the corresponding requirements.

**Sample storage**

The samples are prepared and stored below -80℃.

**Testing Protocol**

Sample preparation

The organoids are cultured in the Matrigel to a stable growth state, and then they are mechanically pipetted out of the Matrigel. The mixture is collected in a centrifugal tube, the organoids are collected by centrifugation, and the supernatant is discarded.

Extraction of DNA

Extract the genomic DNA of organoids according to the instructions of the cell DNA extraction kit.

DNA sequencing

Send the organoid DNA samples and original tumor tissues to institutions qualified for clinical genetic testing for 1^st^ generation sequencing or 2^nd^ generation sequencing testing, or the genetic loci will be tested by ARMS method or ddPCR method.

**Results analysis**

Analyze the mutant loci and compare the concordance between organoid sequencing and original tumor tissue sequencing results.

## Abbreviations

DMSO: Dimethyl Sulfoxide
DNA: Deoxyribonucleic Acid
HBV: Hepatitis B Virus
HCV: Hepatitis C Virus
HIV: Human Immunodeficiency Virus
H&E: Hematoxylin and Eosin
PBS: Phosphate Buffer Saline
STR: Short Tandem Repeat
